# Human Clinical Trials of Probiotics: A Call to Improve Study Design, Conduct, and Reporting

**DOI:** 10.1016/j.advnut.2026.100691

**Published:** 2026-06-22

**Authors:** Kevin Whelan

**Affiliations:** Department of Nutritional Sciences, King’s College London, London, United Kingdom

Probiotics are live microorganisms that, when administered in adequate amounts, confer a health benefit to the host [1].

Fermented cultures have been used for culinary and health purposes for centuries; however, research on their impact on human health only began in the early 20th century. Elie Metchnikoff observed that Bulgarians with high intakes of fermented milk had greater longevity, meanwhile, further human experimental studies at the Institut Pasteur confirmed the effects of probiotics on intestinal metabolism [2]. Since these early ecological studies and uncontrolled trials, there have been many more observational and experimental studies, including multiple randomized controlled trials (RCTs), and there is now extensive evidence for the effectiveness of probiotics in preventing or treating a number of disorders.

The Cochrane Library currently lists ≥34 systematic reviews of RCTs specifically in relation to probiotics. Not all show evidence for effectiveness, although pooled evidence shows probiotics to be effective in a range of conditions across the life course and in a range of settings, including for the prevention of necrotizing enterocolitis in very preterm infants [3], acute upper respiratory tract infections in children and adults [4], and ventilator-associated pneumonia in critical illness [5]. However, numerous of these systematic reviews report that many of the included RCTs incorporated design elements at “high risk of bias” or had “unclear risk of bias” due to poor reporting. Limitations of probiotic study design and reporting are not just an issue for researchers and the probiotic industry; they likely limit clinical recommendations and utilization. Questionnaire surveys of health professionals [6] and consensus opinions of experts [7] indicate varying knowledge and confidence in the existence and quality of the research evidence for probiotics and that this is related to variable and often limited use in practice.

Here exists the paradox: there are a large number of RCTs of probiotics in a range of health and clinical settings, but of variable quality in design, conduct, and reporting that limits their application to practice.

The PRObiotic human study Design And REporting (PRODARE) are new recommendations, published in the current issue of *Advances in Nutrition*, that aim to improve the design, conduct, and reporting of probiotic human trials [8].

There are already multiple recommendations for how RCTs should be conducted (e.g., Good Clinical Practice) and reported [e.g., Consolidated Standards of Reporting Trials (CONSORT)] [9], and therefore, why are specific recommendations required for probiotics?

First, probiotic science is in its relatively infancy, especially within the clinical setting, where human studies can involve novel probiotic strain(s) or novel clinical indications. Second, rapid expansion in techniques to measure outcomes, including genomic analysis of the microbiome and metabolomic measurement of function, has improved the capacity to explore mechanisms of action. As a result of both, various study designs beyond just RCTs can be adopted depending upon the stage of hypothesis development, novelty of the probiotic or clinical indication, or nature of the research question (e.g., mechanisms or efficacy). Third, CONSORT guidelines require that the intervention is described in sufficient detail to allow replication [9], which can be somewhat simpler for an approved pharmaceutical agent delivered via a specific route and in a prescribed dose. However, there are crucial additional details required for probiotic interventions, such as information on the delivery method (e.g., supplement, powder, fermented milk, probiotic yogurt, and fortified food), adherence, as well as quality control procedures prior to or during the trial that may impact probiotic viability, including probiotic production, storage, and handling.

The PRODARE recommendations were initiated by the Probiotics Task Force of the International Life Sciences Institute Europe, which assembled a panel with diversity of professional experience, including academics, clinicians, and industry research and development representatives, but all with expertise in probiotic research ranging from infants, adults, to older people [8]. The panel posed specific questions, reviewed previous human research recommendations, and hosted a workshop to develop a consensus on expert-informed recommendations for probiotic trials, although formal guideline development processes adopting panel voting or the Delphi technique were not followed.

PRODARE provides 7 general recommendations relating to the population (age, diet, and lifestyle), intervention (strain, dosage, and live cell count), outcomes (core outcome sets), and study design (washout periods, prevention of cross-contamination, and trial registration). These are followed by a series of recommendations for 5 different study designs: exploratory studies, pilot studies, RCTs, real-world studies, and observational studies. For each of these, there are specific recommendations on conduct and reporting and, importantly, on which generic reporting recommendations (e.g., CONSORT and STROBE) should be used in conjunction with PRODARE. It is essential these generic reporting guidelines are also used, as understandably PRODARE do not address generic, but crucial, aspects of study design such as sample size calculations, randomization, blinding, and recruitment (including retention and withdrawal). PRODARE also provides an excellent researcher algorithm to align research objectives with study design and reporting outcomes [8].

This is not the first attempt to propose recommendations for the conduct and/or reporting of probiotic human trials. Previous recommendations have aimed to improve overall reporting of the intervention [10], and more recently, specifically how such trials should account for diet and nutrition [11]. These two can be used in conjunction with PRODARE.

Adverse events and safety are given some attention in PRODARE, with recommendations that these may be the focus of exploratory or pilot studies and that safety should be reported in RCTs. Poor adverse event reporting is common in human trials of probiotics. In a systematic review of 384 probiotic RCTs, the majority failed to define or adequately report the number of adverse events or serious adverse events, and where they were reported, they were often using generic descriptions [12]. Reporting of adverse events is a requirement in CONSORT guidelines and so should be covered there; however, due to the evidence of poor adverse event reporting in probiotic research, PRODARE missed an opportunity to specify exact reporting requirements for adverse events.

PRODARE will only improve the design, conduct and reporting of human probiotic trials if they are used. This is a call to the probiotic and wider research community to adopt these excellent recommendations. Academic, clinical, and industry researchers should use PRODARE in designing, conducting, and reporting their probiotic research. Journal editors should request that manuscripts are submitted with evidence of reporting against the 7 recommendations specific to probiotics alongside the recommendations specific for each of the 5 study designs. Finally, peer reviewers should review manuscripts against these recommendations and request amendments where they are not met. PRODARE will therefore hopefully lead to improved design, conduct, and reporting of probiotic human studies and thus hopefully to the potential for probiotic science to improve human health and prevent and treat disease.

## Author contributions

The sole author was responsible for all aspects of this manuscript.

## Funding

The author reported no funding received for this study.

## Conflict of interest

KW has received research grants related to diet and gut health and disease from the Almond Board of California, Danone, and International Nut and Dried Fruit Council and has received speaker fees from Yakult. KW is the holder of a joint patent (PCT/GB2020/051604) to use volatile organic compounds as biomarkers in irritable bowel syndrome for which there is currently no commercial product on the market. KW receives royalties from Wiley Publishing in relation to academic textbooks on Advanced Nutrition and Dietetics.
